# Locating macromolecules and determining structures inside bacterial cells using electron cryotomography

**DOI:** 10.1016/j.bbapap.2018.06.003

**Published:** 2018-09

**Authors:** Charlotte E. Melia, Tanmay A.M. Bharat

**Affiliations:** a Sir William Dunn School of Pathology, University of Oxford, Oxford OX1 3RE, United Kingdom; b Central Oxford Structural and Molecular Imaging Centre, University of Oxford, Oxford OX1 3RE, United Kingdom

**Keywords:** Cryo-ET, Tomography, Bacteria, Sub-tomogram averaging, Microbiology

## Abstract

Electron cryotomography (cryo-ET) is an imaging technique uniquely suited to the study of bacterial ultrastructure and cell biology. Recent years have seen a surge in structural and cell biology research on bacteria using cryo-ET. This research has driven major technical developments in the field, with applications emerging to address a wide range of biological questions. In this review, we explore the diversity of cryo-ET approaches used for structural and cellular microbiology, with a focus on *in situ* localization and structure determination of macromolecules. The first section describes strategies employed to locate target macromolecules within large cellular volumes. Next, we explore methods to study thick specimens by sample thinning. Finally, we review examples of macromolecular structure determination in a cellular context using cryo-ET. The examples outlined serve as powerful demonstrations of how the cellular location, structure, and function of any bacterial macromolecule of interest can be investigated using cryo-ET.

## 1 Introduction

The advent of high-resolution cellular imaging methods has opened new avenues for investigating biological processes in their native environment. Now, the role of macromolecules in driving cellular reactions can be investigated in unprecedented detail using a variety of imaging methods available to the modern cell biologist. These methods can be used to uncover the spatial arrangements of molecules in biological specimens, and reveal how they coordinate fundamental physiological processes. The choice of imaging approach is dependent in the first instance on the biological question, but must also account for the properties of the specimen being investigated. Eukaryotic cells are typically several microns in size, and thus many eukaryotic cellular processes can be readily monitored using light microscopy techniques. On the other hand, bacterial cells are often an order of magnitude smaller than eukaryotic cells and thus present different challenges for cellular imaging. Studying biological processes inside bacterial cells generally requires higher spatial resolution, since processes typically occur over a much smaller scale (<1 μm).

An imaging method that is ideally suited to studying bacterial cells is cryo-EM (electron cryomicroscopy or cryo-electron microcopy). Cryo-EM is a unique imaging tool for the visualization of unstained biological material at high spatial resolution [1]. The specimen under investigation is prepared in a thin layer of vitreous ice, where its ultrastructure is preserved. This frozen hydrated specimen is imaged using an electron microscope operating at liquid nitrogen temperature. Macromolecules, organelles or cells preserved in the vitreous ice layer scatter the incident electron beam and an image of the specimen is recorded using film, CCD cameras or more recently, high efficiency direct-electron detectors [2]. To obtain high-resolution, three-dimensional (3D) snapshots of unique objects such as bacterial cells, one application of cryo-EM, known as cryo-ET (electron cryotomography or cryo-electron tomography) may be employed [3]. In cryo-ET, multiple cryo-EM images of the specimen are recorded at different tilt angles with respect to the electron beam. These images are then computationally combined to obtain a 3D density of the specimen in a reconstructed volume called a tomogram. Next, repeating densities inside tomograms can be averaged together for macromolecular structure determination in a method known as sub-tomogram averaging [4]. Cryo-ET combined with sub-tomogram averaging can thus support structural biological investigations in a cellular context.

The signal to noise ratio of cryo-EM data is inherently limited by specimen thickness, since the number of inelastic scattering events, or ‘noisy’ electrons increase with increasing thickness. During cryo-ET data collection, the effective thickness of the slab-like specimen increases significantly at high tilt angles. Thin (< 1 μm) bacterial cells are therefore more suited to cryo-ET imaging than eukaryotic cells, because the entire organism can be imaged using this method. Applied in this way, cryo-ET can provide not only structural information, but important contextual information about any macromolecule of interest, in particular its organisation or interactions with other molecules within the cell. Imaging unstained, whole, frozen-hydrated cells provides unique interpretative clarity when compared with methods that require extensive interventions ahead of data collection, such as chemical fixation and heavy metal staining.

As evidenced by the wealth of recent articles on the subject, cryo-ET is rapidly becoming a fundamental technique for macromolecular structure determination, particularly of molecules in their cellular context [[5], [6], [7], [8]]. Pioneering developments in the cryo-ET field have been driven by the increasing demand for visualizing macromolecules inside bacterial cells using electron microscopy. With the rise of antimicrobial resistance, understanding fundamental bacterial cell biology and bacterial pathogenicity has come to the forefront of public awareness, placing renewed focus on cryo-ET as a tool to investigate bacterial specimens. This is because cryo-ET provides the unique capability to resolve structures of macromolecules from pathogenic bacteria in a near-native state; *in situ* within cells or on bacterial membranes. Cellular structures derived from cryo-ET represent native conformations of macromolecules found within bacteria, and also shed light on the mechanisms responsible for bacterial evasion of antibiotics, which can inform the rational design of novel strategies to circumvent antimicrobial resistance [9].

In this article, we review recent studies that describe spatial mechanisms in bacterial physiology, placing a special focus on strategies used to localize macromolecules inside cells with the end goal of sub-tomogram averaging and structure determination. We include a discussion of supporting techniques such as cryo-fluorescence microscopy, which provide a crucial navigational guide within subcellular volumes for targeted cryo-ET imaging, as well as sample thinning strategies for collecting high-quality cryo-ET data. Once the location of the target macromolecule is ascertained, cryo-ET data can be used for sub-tomogram averaging, where information about the secondary structure of macromolecules may be resolved. Studies using this approach have revealed fascinating details about the functional states of complexes in their cellular context, which are otherwise inaccessible through other biochemical methods. We thus provide a comprehensive overview of modern cryo-ET workflows as they have been applied to the investigation of bacterial cells, assessing the associated challenges and the approaches used to overcome them. The studies described demonstrate how cryo-ET has been and will continue to be a powerful technique for probing the ultrastructure of whole bacterial cells, for locating molecules of interest with high-spatial resolution, and for structure determination, which together provide a unique glimpse into the subcellular world with near atomic resolution.

## 2 Locating target macromolecules in bacterial cells

### 2.1 Identifying macromolecules by direct observation using cryo-ET

A critical step in the cryo-ET workflow is the identification and localization of macromolecules of interest within tomographic volumes of the specimen. Sub-tomograms of the target macromolecule can then be extracted and averaged for structure determination. The problem is that cryo-EM imaging produces greyscale (black and white) images. As a result, macromolecules of interest are often obscured by, or indistinguishable from their crowded environment. Many cryo-ET studies have therefore focussed either on large proteinaceous assemblies such as sheets or filaments, or on molecules associated with defined cellular locations like membranes or the nucleoid.

**Fig. 1:**
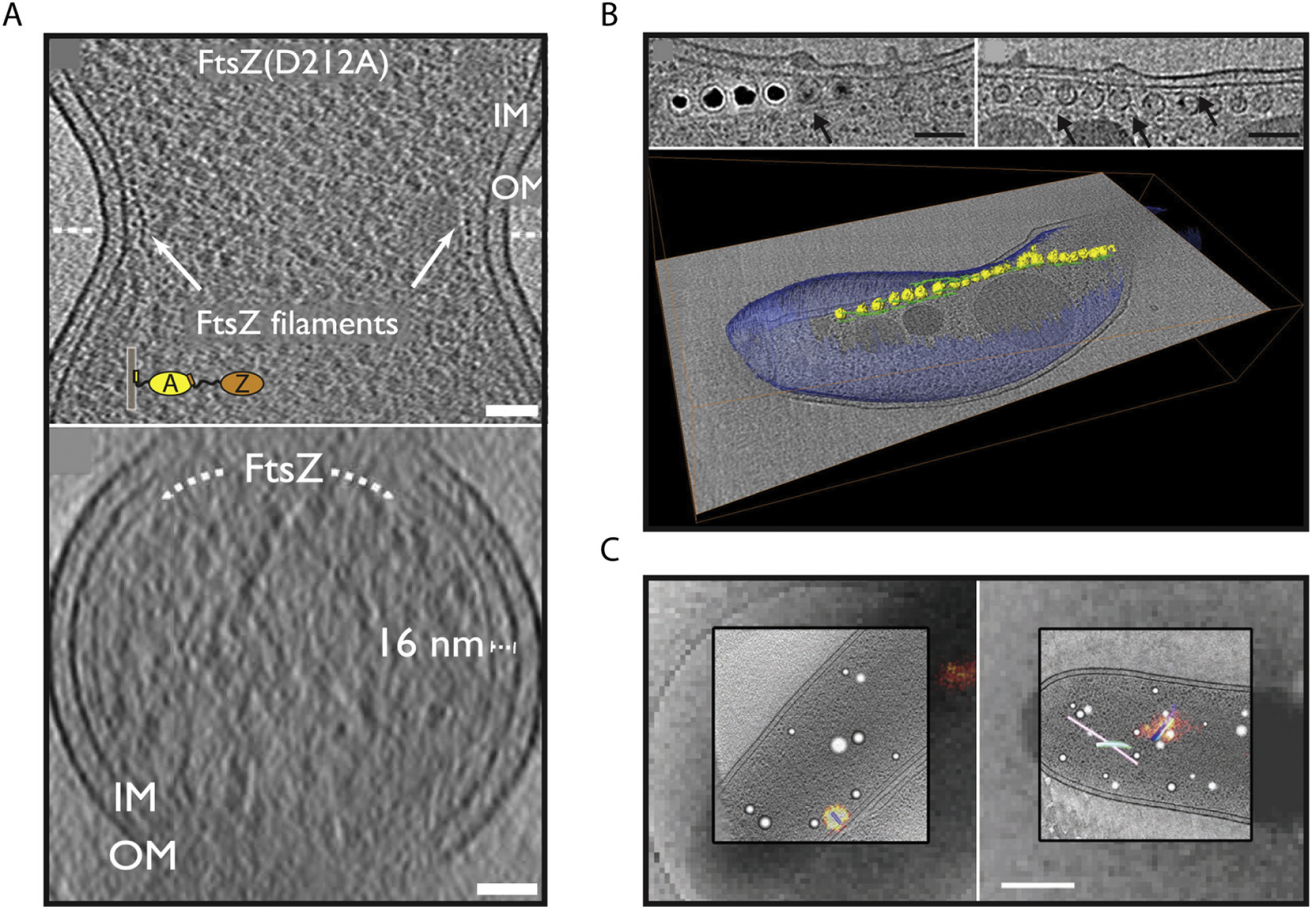
(A) Slices through tomograms of *E. coli* cells slightly over-expressing FtsZ(D212A) protein. A cross-section of FtsZ filaments (white arrows) can be seen at the *E. coli* membrane constriction site (upper). Filaments can be seen across the longitudinal axis at a distance of ~16 nm from the bacterial inner membrane (lower). IM = inner membrane, OM = outer membrane. Scale bars; 50 nm. Adapted from [12]. (B) Magnetosome chain growth in *M. magneticum* in the presence (left) and absence (right) of iron. Segmentation of the latter condition is shown (lower) highlighting magnetosomes (yellow) and their associated filaments (green) and the cellular context (blue). Arrows indicate long cytoskeletal filaments. Scale bars; 100 nm. Adapted with permission from [15]. (C) Single tomographic slices from high resolution cryo-ET reconstructions of *M. xanthus*, with object segmentations and overlaid cryo-PALM signal. A very short loaded T6SS structure with a ‘baseplate’ attached to the membrane (left) and a distinctive bent T6SS structure (right) are shown. Segmentations show different tubular structures (blue, green, pink) and spherical granules (white). Scale bar; 400 nm. Adapted with permission from [19].

One area of intense research is the study of protein and membrane dynamics involved in bacterial cytokinesis. Bacterial cytokinesis is mediated by the bacterial homologue of tubulin known as FtsZ [10]. A ring of FtsZ molecules assembles at the mid-cell, driving membrane invagination and the formation of two daughter cells. Arc-like filaments were observed at the *Caulobacter crescentus* mid-cell [11]. Upon over-expression of FtsZ, the abundance and length of these filaments increased and filaments persisted even in the presence of an MreB (bacterial actin) inhibitor, demonstrating that the observed filaments were not comprised of MreB, but rather FtsZ [11]. Together, these experiments confirmed the identity of FtsZ filaments at the mid-cell, and demonstrated that arc-like FtsZ filaments mediate cytokinesis. Another study conducted more recently using direct-electron detectors and the latest generation microscopes revealed that complete FtsZ-rings are formed at the mid-cell in *C. crescentus* and *Escherichia coli* during cytokinesis [12] (Fig. 1A). These cryo-ET data from cells combined with *in vitro* experiments led to a model of sliding FtsZ filaments in a ring driving membrane constriction at the mid-cell [12]. Finally, data from *C. crescentus* and *Proteus mirabilis* showed that short FtsZ filaments can cause asymmetric cell envelope constriction at the beginning of cytokinesis [13]. The extension of these short filaments would lead to the formation of a ‘Z'-ring driving cell division. These studies on bacterial cytokinesis were made possible by increasing the abundance of FtsZ molecules in cells relative to the natural state, and by comparing FtsZ filaments found in different organisms (*C. crescentus*, *E. coli* and *P. mirabilis*). Together, these strategies led to an unambiguous identification of FtsZ inside bacterial cells and provided important insights into the fundamental process of cell division.

A related strategy to locate molecules in cells using cryo-ET is to either delete the gene of interest completely, or perturb its copy number in a controlled manner, leading to distinctive changes in the ultrastructure of the cell. By comparison with wild-type cells, the location of the target molecules may be inferred. The success of this strategy depends on the possibility to identify the structures of interest (or lack thereof) after genetic manipulation. Despite this requirement, this approach is extremely useful for macromolecular identification of larger protein assemblies (*e.g.* bacterial secretion systems or cytoskeletal proteins). A dramatic example of this strategy is provided by studies on membranous organelles present in *Magnetospirillum* bacteria called magnetosomes. Magnetosomes are flanked by a network of actin-like proteins called MamK. MamK protein filaments appear to organise magnetosomes into linear chains that provide magnetic orientation to cells [14]. Unambiguous evidence for this scenario was provided by comparing wild type and *mamK* deleted cells using cryo-ET [14, 15]. Without MamK filaments, long chains of magnetite particles (Fig. 1B) could not assemble in cells, and patches of particles distributed unevenly throughout the cell were observed instead [14, 15].

Another cellular process mediated by cytoskeletal actin-like proteins is plasmid segregation by the ParMRC system. ParM is an actin-like protein that pushes replicated low-copy number plasmid sisters to opposite ends of dividing cells. The tips of ParM filaments are attached to an adaptor protein called ParR, which in turn binds to a centromeric *parC* DNA sequence present on the plasmid [16]. Insights into the mechanism of plasmid segregation were furnished by imaging *E. coli* cells containing plasmids at different copy numbers, each encoding the *parMRC operon* [17]. Long doublets of ParM filaments were observed in the cytoplasm, roughly aligned with the long axis of the cell. The numbers of doublets observed per cell correlated perfectly with the expected copy numbers of plasmids, strongly suggesting that ParM doublets form a spindle that allows faithful plasmid segregation in cells [17]. Thus, *in situ* cryo-ET studies indicate that each replicated plasmid pair generates their own spindle containing a doublet of ParM filaments. This ensures that equal copies end up on opposite ends of the cell, eliminating the requirement for DNA congression or checkpoints which are the hallmark of eukaryotic mitosis [17].

The examples described in this section illustrate that while cryo-ET is a visual, image-based technique which, when taken in isolation, may seem merely descriptive, it can provide profound mechanistic insights into bacterial cell biology. Comparing different perturbed states of the system under observation allows researchers to glean mechanistic understanding of dynamic biological processes. These studies are particularly suited to study macromolecules that oligomerise into an easily identifiable shape (*e.g.* filaments or sheets) in cells. In the examples presented above, a common approach used is modification of the copy number of the macromolecule of interest, and comparison of the resulting cellular ultrastructure with that of wild-type cells to understand the location and function of the macromolecule.

### 2.2 Locating molecules using correlative Cryo-LM and Cryo-ET

Intrinsic, unambiguous shapes or other defining features to identify macromolecules of interest within tomographic volumes are often lacking, particularly where little prior information about the macromolecular target is available. Furthermore, the macromolecule may be found only in a subpopulation of cells, making its identification challenging even with the over-expression or deletion strategies described above. One approach to overcome this problem is to utilise fluorophores whose fluorescent properties can be preserved at cryogenic temperatures [18]. Fluorescently tagged macromolecules can be imaged in a cryo-LM (cryo-light microscopy) step prior to of cryo-ET, and in this way, provide a fluorescent map to guide researchers to suitable regions for tomography.

Performing fluorescent light microscopy at low temperatures poses a number of challenges. To ensure the working temperature is within lens tolerance, cryostage systems for LM typically incorporate objectives with a long working distance, which limits the achievable numerical aperture (NA) and final image resolution. Additionally, cryo-LM adds an extra cryotransfer step to the workflow, which increases the risk of ice contamination and/or devitrification. Furthermore, bacterial cells are extremely small, and high-resolution LM images are required for sub-cellular localisation of any fluorescent signal. Super-resolution LM approaches, and their associated benefits, are now being ported into cryo-LM systems [[19], [20], [21]]. Techniques such as single-molecule localisation microscopy (SMLM) circumvent the resolution limits of conventional long working-distance lenses used for cryo-LM and improve upon even optimal diffraction-limited imaging to achieve resolutions of <100 nm. With the improved resolution in cryo-LM, fiducial markers that are visible in both cryo-LM and cryo-EM may be used to correlate features and localize fluorescent signals in the electron microscope with high precision (<200 nm) in a combined process known as cryo correlative light and electron microscopy (cryo-CLEM).

Cryo-LM/cryo-ET workflows have been successfully applied to study a variety of bacterial processes. The vegetative growth phase of *Streptomyces* is a dramatic process involving the formation of long hyphae separated by cross-walls that exclude nucleoids. The distance between cross walls is several micrometers, thus requiring a modest resolution in cryo-LM for visualization and unambiguous correlation. A workflow to investigate this process using cryo-CLEM on *Streptomyces* cells was developed and applied to probe the underlying ultrastructure of cross walls [22]. A fluorescent membrane stain was used for cryo-LM imaging, which revealed the presence of long tubular membrane invaginations in cryo-ET [23]. The membrane invaginations were accompanied by many small vesicles, which were readily observable in reconstructed tomograms (cryo-ET) but not in cryo-LM. This example illustrates the power of fluorescence microscopy as an informative guide for targeted high-resolution cryo-ET imaging.

However, features of interest in bacterial cells are rarely several micrometers apart, and higher resolution is typically required in cryo-LM to unambiguously localize macromolecules in each diffraction-limited fluorescent spot. This was precisely the case in the study of a dynamic Type-VI secretion system (T6SS) in *Myxococcus xanthus* cells [19]. Ordinary cryo-LM produced a broad, low-resolution fluorescent signal in the crowded interior of *M. xanthus* cells, which could potentially have been assigned to one of a variety of candidate filamentous structures present within the cells [19]. To get around this problem PALM (photoactivated localization microscopy), a SMLM approach that uses laser activation and inactivation of photoactivable fluorophores, was applied. Out of many fluorophores that were tested, one fluorophore PA-GFP (photoactivable green fluorescent protein) maintained its photoactivable behaviour in cryo conditions [19], and type-VI structures tagged with PA-GFP were studied using correlated cryo-PALM-cryo-ET. Type-VI structures in a variety of conformations and constitutional states were located using this approach and imaged at high-resolution using cryo-ET [19], including previously unidentified structures that may represent the forming and disassembling T6SS (Fig. 1C). These approaches, which circumvent challenging resolution boundaries, pave the way for future studies of structure and function over nanoscale distances in bacterial cells, and provide a powerful way to locate molecules inside bacteria.

### 2.3 Macromolecular tags for EM

Performing cryo-LM prior to cryo-EM is nevertheless cumbersome, and even the best resolutions attainable by cryo-LM can severely limit the feasibility of some projects. To combine the spatial resolution of EM with the specificity of a tag for macromolecule localization within cellular tomographic volumes, ideally the tag should be visible directly in EM. Transmission EM images (*i.e.* ordinary cryo-EM images) do not have ‘colour’ and the intensities in images are roughly correlated with the quantities and properties of scattering material in the specimen. Direct EM tags must therefore be significantly more electron dense than the surrounding biological material. The most common EM tags used thus far in the field have relied on a metal loaded protein such as ferritin [24] or metallothionein [[25], [26], [27]]. This method of tagging also requires that the metal ion of interest is loaded into the cells prior to cryo-EM sample preparation. For bacterial cells, high metal concentrations are often toxic, rendering this approach difficult to implement in practice.

One proof-of-principle study that succeeded in fulfilling most of these requirements in bacteria used bacterial ferritin (FtnA) overexpression in a *fur* knockout *E. coli* strain. Without the Fur protein repressor, the cellular iron concentration is no longer tightly regulated, facilitating metal loading of FtnA molecules [24]. In tomograms, loaded ferritin molecules were clearly observed in the cytoplasm of *E. coli* cells. Upon fusion of a membrane targeting sequence to FtnA, the ferritin signal localized instead to the bacterial membrane [24]. Finally, the chemotaxis response regulator CheY, and the cell division protein ZapA, were tagged with FtnA leading to the striking accumulation of ferritin density at the cell poles and at division septa respectively [24]. Similar tagging approaches will be important in the future, adding significant explanatory power to cryo-ET data and facilitating high-precision localisations of macromolecules in cells. The development of a widely-applicable tag for cryo-EM, similar to GFP for LM, remains an outstanding goal for the field.

## 3 Sample thinning approaches to support cryo-ET investigations

Many bacteria including *B. subtilis* and standard lab strains of *E. coli* are too thick for high-resolution cryo-ET, especially when the features of interest are located deep in the interior of the cell. In these cases, there are several thinning approaches available that convert the specimen into a viable target for cryo-ET data collection. These approaches are rapidly gaining importance as the field progresses towards tackling novel and ever more ambitious biological questions, and will be discussed in some detail. For many samples, thinning is critical for the success of *in situ* structural biology as it facilitates both the identification of molecules within cells, and supports high-resolution sub-tomogram averaging.

### 3.1 Thinning by controlled lysis of cells

Prior to vitrification, cells can be artificially thinned by a gentle lysis step. The lysis process produces thinner ‘ghost’ cells that lose some of their rigidity, allowing them to flatten during the blotting and plunge-freezing protocol in cryo-EM grid preparation. While lysis itself is relatively gentle, it nevertheless represents significant disruption to living cells prior to their cryo-fixation, and the potential for artefactual results must be considered. This approach was developed for visualizing native *E. coli* membranes by triggering lysis upon over-expression of a phage lysis gene [28]. A tightly controlled plasmid expression system was used to ensure that the lysis gene was expressed only when required. Partially lysed *E. coli* cells were obtained upon induction, which efficiently flattened during grid preparation allowing high-resolution cryo-ET imaging and sub-tomogram averaging of bacterial chemoreceptor arrays [28]. Imaging chemoreceptor arrays has similarly been achieved in thick *B. subtilis* cells using Lysozyme treatment [29] and in *E. coli* cells using penicillin treatment [30]. Both of these approaches lead to partial cell lysis and cell flattening, thus supporting high-quality cryo-ET data collection.

### 3.2 Thinning vitrified cells by sectioning in an ultramicrotome

One obvious advantage of thinning the specimen after vitrification is that no genetic or biochemical manipulation is required prior to vitrification, reducing the danger of artefacts and incorrect interpretation of cryo-ET results. To achieve this, there are two major approaches available. In one approach, vitrified specimens may be sectioned using an ultramicrotome operating at liquid nitrogen temperature, where the vitreous sections of cells are deposited on an EM grid. This technique is known as CEMOVIS (cryo-electron microscopy of vitreous sections) [31], and it has been successfully used to image the interior of bacterial cells [32]. Each section of the specimen in theory contains hundreds of cells to choose from for cryo-ET imaging. CEMOVIS also has the potential to support high-precision localization of macromolecules using cryo-CLEM methods [33]. These advantages have been exploited in several CEMOVIS studies conducted on bacterial cells. One such study focussed on the plasmid segregation machinery encoded by the *parMRC* operon [34]. Direct cryo-ET imaging of *E. coli* cells was problematic due to thickness, particularly without the aid of direct electron detectors. CEMOVIS was therefore used to visualize the arrangement of ParMRC spindles in cells [34]. Actin-like ParM filaments were observed in cross-sections of *E. coli* cells containing low-copy number plasmids in the act of being segregated, showing for the first time with high-resolution cryo-EM that a ParM filament spindle mediated this process [34]. No changes in the filaments were observed when an anti-MreB inhibitor was added to the cells, and Fourier analysis of the helical repeat of filaments agreed perfectly with that of purified ParM filaments [34]. Other CEMOVIS studies have concentrated on studying the bacterial cell envelope from a variety of bacteria such as *E. coli, Pseudomonas aeruginosa* and *Mycobacterium smegmatis* [32, 35].

Despite these success stories, there are several disadvantages associated with CEMOVIS. CEMOVIS is labour intensive and sample preparation is relatively low-throughput; only a few grids may be prepared in each sectioning session. Due to the long sectioning process and additional grid transfer steps, ice contamination may build up on the grid, obscuring features of interest. Additionally, due to poor attachment of sections on the grid, cryo-ET data collection is also complicated because of section movement during tilt series data collection [33, 36]. Finally, the most detrimental consequence of cryo-sectioning are due to the action of the diamond knife of the cryo-ultramicrotome [37], which leads to crevassing and compression artefacts on the surface of the section. While there are approaches to minimize these artefacts, high-resolution sub-tomogram averaging is currently not possible from vitreous sections. Therefore, sub-tomogram averaging efforts on vitreous sections have been limited to low-resolution sub-tomogram analysis and two-dimensional fourier analyses [33, 34].

### 3.3 Thinning using a focused-ion beam

With the advent of focused ion beam (FIB) milling, sample thinning can now be achieved in a controlled manner, where regions around the feature of interest are carefully etched away to form a thin lamella or wedge suitable for cryo-ET data collection [38]. This technique is ideal for examining the interior of eukaryotic cells where structures including the nucleus, ribosomes, endoplasmic reticulum and mitochondria can be readily resolved [39]. Sample thinning is also advantageous for bacterial cells, where improved contrast in images leads to higher quality tomograms. A significant advantage of cryo-FIB milling is the relatively low risk of surface artefact generation relative to CEMOVIS sections [40]. Cryo-FIB milling of specimens can in this way enable high-resolution sub-tomogram averaging [41], making this technique extremely attractive for structural cell biology studies on bacteria.

**Fig. 2:**
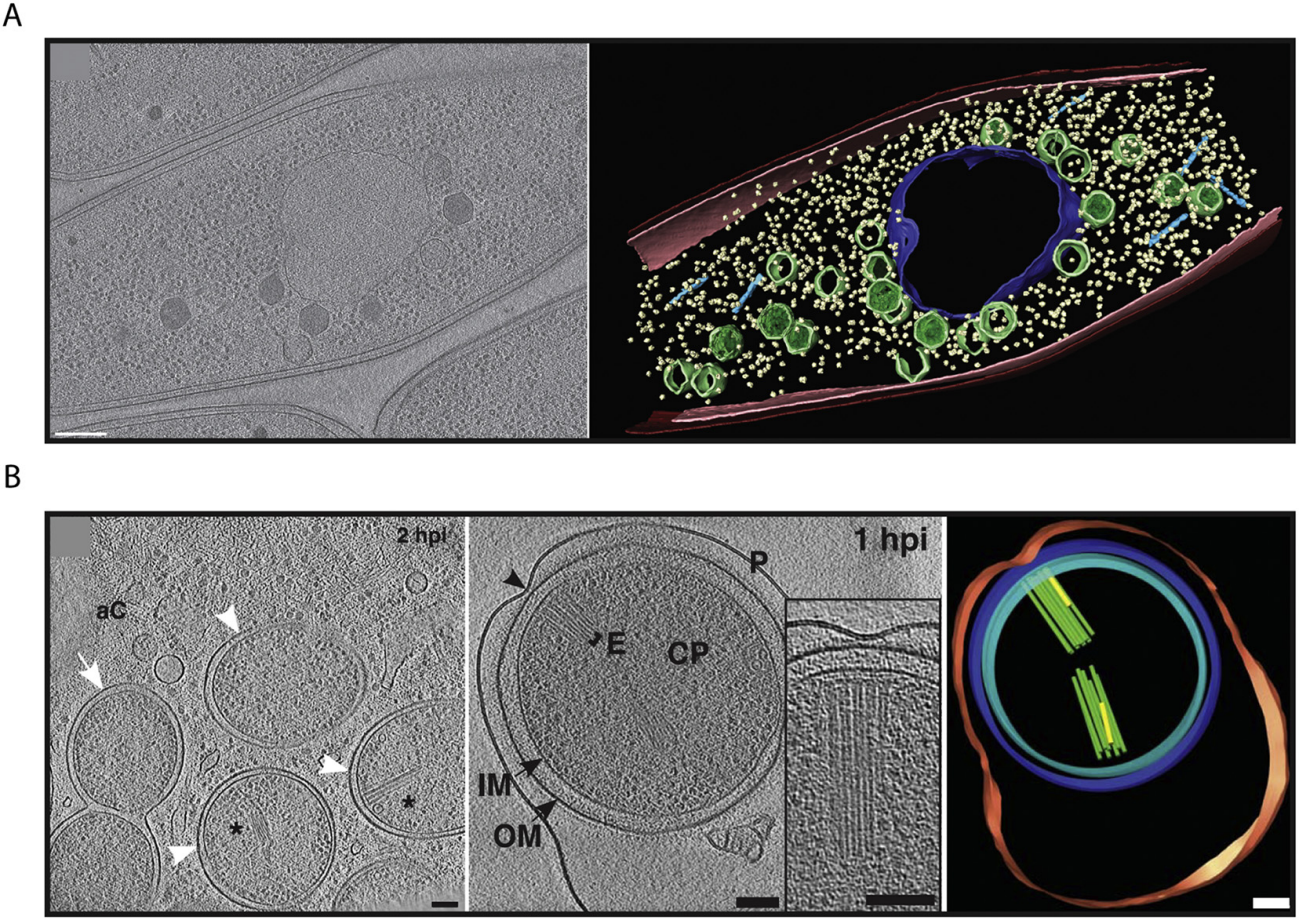
(A) Single slice through a cryo-ET reconstruction of a 201φ2–1 phage-infected *P. chlororaphis* prepared using FIB milling, in which filled (darker) assembled capsids are docked to an apparently contiguous shell during DNA encapsidation. A segmentation of the complete tomogram is shown (right), highlighting the shell (dark blue/purple), capsids (green), cytoplasmic membrane (pink), outer membrane (red), phage tails (light blue), and ribosomes (yellow). Scale bar; 200 nm. Adapted with permission from [42]. (B) *A. asiaticus* bacteria were imaged inside cells using FIB milling and cryo-ET. A representative tomographic slice is shown at later time points post infection (0.5 to 2 h post infection (hpi)) in which amoebophili are found in the amoeba cytosol (aC) (white arrowheads). Amoebophili differentiate into rods (white arrows), although some show signs of degradation (black arrowheads) and do not escape (left). At earlier time points (0.25 h post infection) amoebophili reside within phagosomes, and extended T6SS arrays can be found contacting the phagosome membrane (black arrowhead) (center), highlighted in the corresponding segmentation (right). P/red: phagosome; OM/blue: outer membrane; IM/cyan: inner membrane; CP: cytoplasm; E/green: extended T6SS; yellow: contracted T6SS. Scale bars: 100 nm. Adapted with permission from [43].

This is evidenced by recent landmark studies using FIB-milling of bacterial cells. Phage-infected *Pseudomonas chlororaphis* bacteria were found to contain nucleus-like compartments in which nascent viral capsids are synthesised [42]. FIB milling and cryo-ET of plunge frozen cells revealed that empty assembled phage capsids are tightly attached to the central nucleus-like region. DNA encapsidation occurs at the periphery of the nucleus-like region where filled (darker) capsids are produced [42]. Facilitated by the thinning of the specimen, empty phage capsids, fully assembled phages, phage tails and ribosomes were clearly visible in tomograms [42] (Fig. 2A). Another study on the bacterial T6SS in *Amoebophilus asiaticus* showed bundles of T6SS in the cytoplasm arranged with hexagonal symmetry [43]. Through a combination of cryo-FIB milling and cryo-ET, these T6SS bundles could be resolved in bacteria residing deep within the infected host cell (Fig. 2B). The number of T6SS bundles decreased as the amoebophili escaped the phagosome, but the percentage of contracted (functional) T6SS increased correlating with phagosome escape [43].

The above examples show how the use of cryo-FIB milling and cryo-ET can help in unlocking the unprecedented potential of *in situ* structural biology. However as is the case with CEMOVIS, cryo-FIB milling is an extremely labour-intensive and low-throughput method, although improvements in the field are focused on making the technique more routine [44]. Other considerations include the number of cells available per sample. Only a few target cells can be imaged per grid *via* cryo FIB milling, whereas in a vitreous section obtained by CEMOVIS hundreds of cells can be imaged, dependent on the density of the frozen culture.

## 4 Solving structures of target macromolecules

Important information regarding composition and cellular function is embedded in the high-resolution 3D structure of a macromolecular complex or assembly. Once the cellular location of the macromolecular complex is ascertained, and once the specimen is thin enough for cryo-ET imaging, then sub-tomogram averaging can be used to resolve information at the structural level. For this, copies of macromolecules visible in tomograms are computationally extracted into smaller windowed volumes known as sub-tomograms. These sub-tomograms are then averaged after 3D alignment to reveal higher resolution detail [45, 46]. Sub-tomogram averaging is a powerful technique for 3D structure determination that supports near-atomic resolution refinement [47]. Many high-resolution structures have been reported using sub-tomogram averaging methods [[48], [49], [50], [51], [52]]. However, most of these recent studies have focussed on purified or *in vitro* assembled specimens. Unlike *in vitro* reconstituted samples, structures solved *in situ* retain crucial information regarding the functional state of a target molecule. If a picture of the surrounding cellular region is available, then structural information can be interpreted in conjunction with a wealth of additional contextual information. While this makes whole-cell imaging an attractive option, it comes with significant challenges associated with thick samples (see section on sample thinning above), including sub-tomogram alignment in crowded cellular environments and low signal to noise ratios.

In the sections below we discuss examples of sub-tomogram averaging applied to bacterial macromolecules in a cellular context, either of thin cells or cellular structures, or thicker cells where cryo-ET imaging was nevertheless successful. In all cases, the locations of the target macromolecules were either established previously from live-cell imaging, or were determined using one of the target localisation strategies discussed above.

### 4.1 Sub-tomogram averaging applied to thin cells or cellular structures

One method adopted by researchers to produce high-quality tomograms is to use specialized bacterial strains that produce a mini-cell morphology. These mini-cells are significantly smaller and thinner than wild-type cells, and thus support high-resolution cryo-ET data collection and sub-tomogram averaging. This approach has been used to study bacterial chemoreceptor arrays, which are supramolecular transmembrane machines that localize to the cell poles, allowing cells to sense changes in their extracellular environment and instigate flagellar motility [53]. These arrays were the subject of a detailed cryo-ET study in *E. coli* mini-cells [54]. Aided by the small size of the cells, sub-tomogram averaging maps of the chemoreceptor array were obtained that described the molecular architecture of the array. Six trimers of the chemoreceptor protein (Tsr) formed a hexameric lattice, embedded in a layer of chemotaxis proteins CheA and CheW [54]. CheW is the coupling protein that transduces the chemical signal received by the array to the flagellar motor to control its direction of rotation. CheW was located in close proximity to the chemoreceptor hexamer, thus providing a link for conversion of a chemical signal to directed bacterial motion [54]. A parallel cryo-ET study conducted in *Salmonella enterica* mini-cells along with lysed *E. coli*, *B. subtilis* and *H. hepaticus* cells reported molecular-resolution sub-tomogram averaging maps of the chemoreceptor array into which X-ray structures of the proteins could be docked [29]. The docked model revealed the same hexagonal arrangement in all the organisms studied, and provided a framework for future investigations of the same system. These studies on bacterial mini-cells illustrate the power of tomography and sub-tomogram averaging in understanding cellular physiology with structural biology techniques. When combined with docked atomic structures, even sub-tomogram maps with limited resolution have provided remarkable insights into a highly conserved bacterial macromolecular machine [29].

**Fig. 3:**
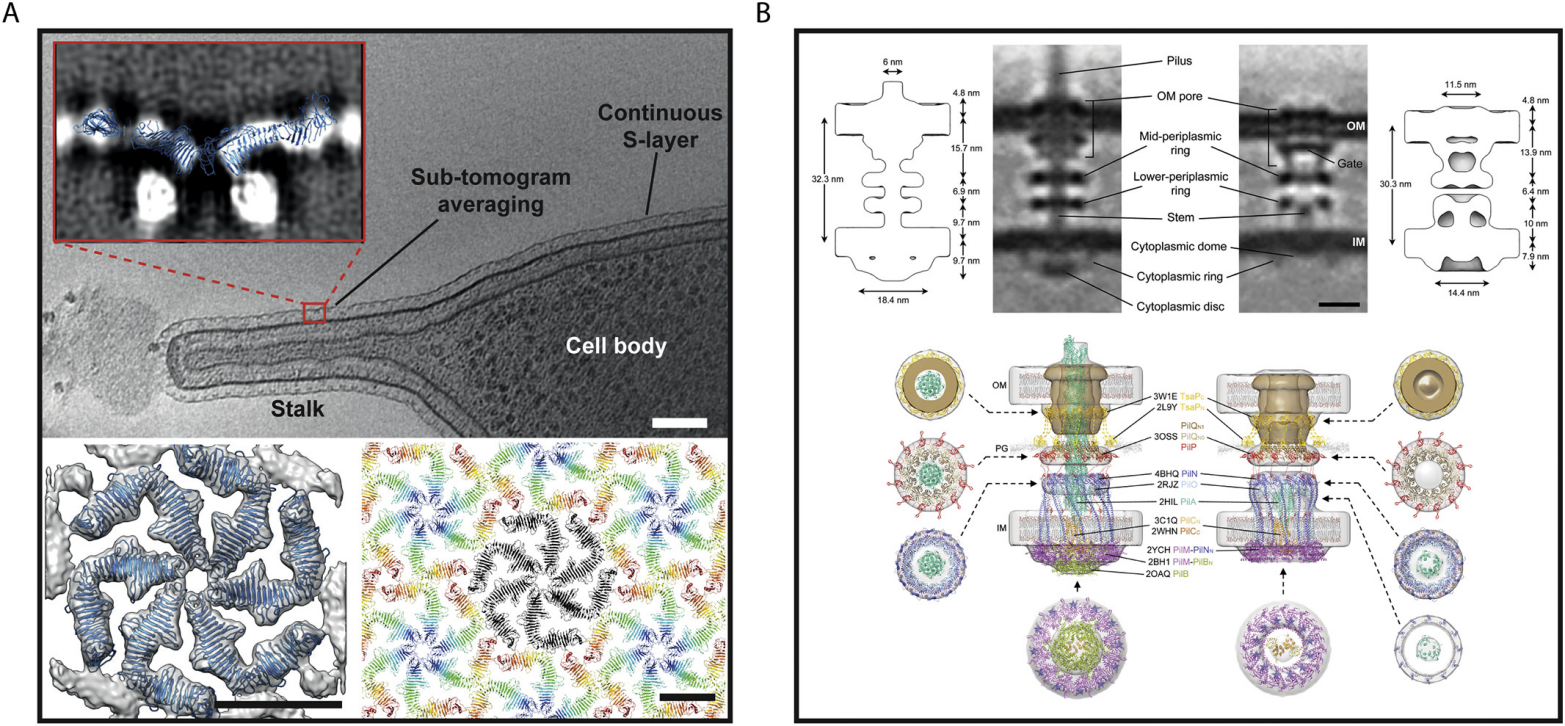
(A) Tomographic slice of a *C. crescentus* cell showing a continuous S-layer between the cell body and stalk (upper). Inset – single slice through the sub-tomogram averaging map generated using cryo-ET, with the fitted S-layer protein hexamer from the X-ray model overlaid. Top view of the outer layer of the sub-tomogram averaging map (grey density) with a fitted X-ray structure (blue ribbon) (left). X-ray crystallography structure of a hexamer:hexamer repeat is shown which resembles the S-layer lattice structure derived from cryo-ET. Scale bars; 100 nm (upper), 10 nm (lower). Adapted from [55]. (B) Subtomogram average slices of piliated (left) and non-piliated (right) T4 PM basal bodies in *M. xanthus*. Hypothetical working models of these structures are shown below, with atomic models of individual T4 PM components overlaid onto the resolved densities, or shown separately (top views). Blue stars on PilM rings indicate the locations of bound PilN N-termini. Scale bar; 10 nm. Adapted with permission from [57].

The advantages of imaging thin structures are illustrated by structural studies of the *C. crescentus* surface layer [55] on a thin (120 nm), elongated, cytoplasmic appendage known as a stalk. Many Gram-negative and Gram-positive bacterial cells are surrounded by a para-crystalline, proteinaceous surface layer (or S-layer), which protects cells from harmful extra-cellular molecules and provides mechanical stability to membranes. *C. crescentus* cells possess such an S-layer bound to the outer membrane [56]. Sub-tomogram averaging of the *C. crescentus* S-layer from cell stalks led to sub-nanometre resolution reconstruction of the S-layer lattice [55]. The arrangement of the repeating hexameric structures that comprise the S-layer was clearly visible in individual cryo-ET slices (Fig. 3A). Secondary structure elements such as α-helices and β-sheets were visible in the sub-tomogram averaging map. Unambiguous fitting of the X-ray atomic structure of the S-layer lattice into the density was performed to reveal atomic level details of the S-layer lattice on the surface of cells [55]. Interestingly, the body of *C. crescentus* was too thick (~500 nm) for sub-nanometre resolution sub-tomogram averaging, demonstrating that targeting thin areas of cells, or obtaining thin cells can greatly facilitate *in situ* structural biology efforts.

### 4.2 Structure determination from whole cells

In many cases it is not possible to obtain a mini-cell containing the macromolecular complex of interest. Mini-cell formation is also undesirable *in situ*ations where it disrupts the physiologically relevant state of the target complex. In such cases, tomographic data from whole cells must be used, where the best achievable resolution from sub-tomogram averaging is limited due to specimen thickness. However, molecular resolution reconstructions are still achievable, providing important architectural information about protein constitution and conformation.

An in-depth study of the Type-IV pilus (T4P) from *Myxococcus xanthus* showed the power of molecular resolution sub-tomogram averaging from whole cells to reveal assembly principles of macromolecular complexes *in situ* [57]. The T4P is a molecular machine that facilitates bacterial adhesion, virulence and biofilm formation. The localisation and orientation of the T4P machinery (T4 PM) at the cell pole facilitates sub-tomogram averaging, since the thickness of cells at the edge is smallest (especially if pili molecules are parallel to the tilt axis during cryo-ET data collection). Using strains with deleted pilus proteins, or strains with fusions of small domains (such as superfolder GFP) to pilus subunits, a series of sub-tomogram averaging maps of the T4 PM were produced at resolutions of 25–40 Å [57]. These mutant T4 PM maps were all compared to the map of the T4 PM from wild-type cells, and by comparing the visible densities the location of critical T4 PM subunits was ascertained [57] (Fig. 3B, upper panel). Positions of pilus subunits were used to build a working model of the entire Type-IV molecular machine, providing unprecedented biological insight into the mechanism and functional architecture of the native complex on the cell surface [57] (Fig. 3B, lower panel).

Macromolecules of interest are not always found on the cell surface, but often buried deep in the interior of bacterial cells where specimen thickness and poor tomogram quality hinder the identification of individual complexes. Here, to boost contrast at low resolutions, tilt series data are often collected with a large applied defoci. A large defocus is detrimental to sub-tomogram averaging applications however, as it results in increasing oscillations of the contrast transfer function at high resolutions. The contrast transfer function describes how, across different resolution ranges, information is transmitted or lost in the electron microscope. In practical terms, a large defocus leads to improvement in the visibility of low resolution features, but high resolution information is more difficult to recover, even with averaging. One way around this problem is to use a phase plate to obtain tomograms close to focus that preserve the low-resolution contrast while potentially supporting higher-resolution refinement in sub-tomogram averaging. Zernike phase-contrast cryo-ET was used to study phage intermediates inside cyanobacterial cells [58]. Compared to ordinary cryo-ET, low-resolution contrast was dramatically improved in phase-shifted images, allowing identification of virus intermediates even in thick parts of the cell [58]. Sub-tomogram averaging allowed the classification of identified densities into procapsids, phages with and without DNA and fully assembled phage particles, leading to a complete phage assembly model [58]. In the last couple of years, the use of an alternative Volta phase plate has become popular [59]. This phase plate has been successfully used to image bacterial cells with cryo-ET, providing a new avenue of approach for structure determination inside cells [59].

## 5 Outlook

**Fig. 4:**
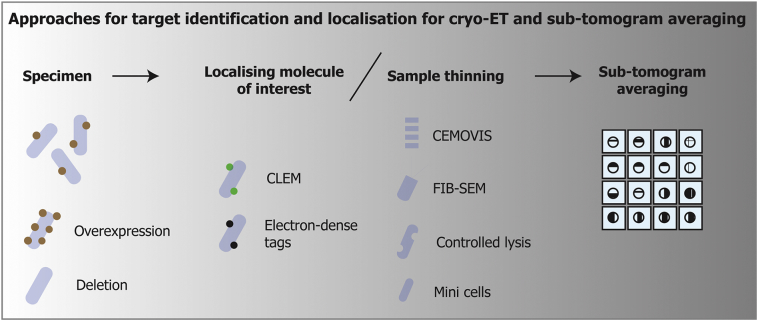
Typical approaches used for *in situ* macromolecular structure determination using cryo-ET and sub-tomogram averaging. The first step is to identify and locate target molecules. This may be achieved using overexpression or deletion and comparison with wild-type cells. The cellular location of molecules can also be ascertained using genetically-encoded tags or cryo-CLEM. Localization and identification strategies can usually be applied either before or after sample thinning, which may be necessary for high quality cryo-ET data collection. Localized macromolecules in thinned specimens can then be imaged using cryo-ET, and densities corresponding to the macromolecule can be used for sub-tomogram averaging refinement and structure determination.

We hope that it is apparent from the preceding text that the potential for structural cell biology studies in bacteria using cryo-ET is huge. While the developed workflows for macromolecular identification and imaging include a range of possibilities for accessing and tagging targets of interest (Fig. 4), we expect that technologies that help to identify macromolecules inside cells will continue to be developed. One of the ultimate goals of cryo-EM method development will be to develop a genetically encoded tag that would be visible in EM, and have widespread applications, much like GFP in optical microscopy. An alternative method for macromolecular identification inside cells that was not discussed in detail is template matching [60]. With higher quality cryo-EM images and tomograms, computational template matching approaches may become more powerful and offer a viable solution for many projects.

The potential for resolving protein structures in their cellular context represents an exciting bridge between the worlds of cellular and structural biology. We expect that through the use of cryo-FIB milling and other thinning approaches, sub-tomogram averaging will start playing a central role in cellular structural biology, although there remains a need to develop sub-tomogram averaging refinement procedures in low-signal to noise ratio environments inside cells [39, 61, 62]. Lines of research that are currently beyond reach will be advanced by the successful development of these and other cryo-ET methods.
